# Current Approaches to Risk Assessment and Prevention of Preterm Birth—A Continuing Public Health Crisis

**DOI:** 10.31486/toj.20.0005

**Published:** 2020

**Authors:** Joseph R. Biggio

**Affiliations:** System Co-Chair of Women's Services, Ochsner Clinic Foundation, New Orleans, LA and Clinical Professor of Obstetrics and Gynecology, The University of Queensland Faculty of Medicine, Ochsner Clinical School, New Orleans, LA

**Keywords:** *17 alpha-hydroxyprogesterone caproate*, *cerclage–cervical*, *cervical length measurement*, *premature birth*, *progesterone*

## Abstract

**Background:** Preterm birth remains a major cause of neonatal morbidity and mortality. Several potential pathways and pathophysiologic processes can lead to preterm birth, complicating efforts to screen for the risk of preterm birth and making implementation of prevention strategies difficult.

**Methods:** Based on a review of the literature, this article addresses screening strategies for preterm birth risk stratification and interventions for preterm birth prevention.

**Results:** In women with a history of a prior spontaneous preterm birth, cervical cerclage placement in the setting of short cervix reduces the rate of recurrent spontaneous preterm birth. Weekly injections of 17-hydroxyprogesterone caproate (17-P) have been used as standard treatment for the prevention of recurrent preterm birth since 2011. However, results of a replication study of 17-P published in 2020 have raised questions regarding the effectiveness of this drug, and it is under review by the US Food and Drug Administration. Among women with no history of preterm birth, cervical length appears to be the best predictor of risk for preterm birth in asymptomatic women. In women with a cervical length <25 mm, vaginal progesterone has been demonstrated to reduce the risk of preterm birth.

**Conclusion:** Strategies including cervical length screening, vaginal progesterone administration, cervical cerclage placement, and, potentially, 17-P administration may help reduce rates of preterm birth when used in the appropriate patient populations. Development of protocols for patient evaluation and risk stratification will help identify patients at highest risk for preterm birth and allow use of the best available therapeutic interventions.

## INTRODUCTION

Preterm birth remains the most common cause of neonatal morbidity and mortality in the United States.^1-3^ Rates of preterm birth in Louisiana are among the highest in the country, with rates in some parishes approaching those of developing countries. While the rate of preterm birth declined nationally from 2007 to 2014, the rate rose to 9.93% in 2017.^4-7^ In Louisiana, the rate of preterm birth at <37 weeks of gestation was 12.7% in 2017. Other states in the southeastern United States have similar challenges with high rates of preterm birth.^6,7^

Although the rate of preterm birth has risen, some interventions have been demonstrated to have a potential role in reducing the likelihood of recurrent preterm birth in women with prior preterm births. Even with these interventions, however, the impact on the overall rate of preterm birth is minimal because 90% of deliveries prior to 34 weeks of gestation and 85% of deliveries prior to 37 weeks of gestation occur in women with no previous history of preterm birth.^8-10^ Therefore, other strategies are needed to identify women at highest risk in the absence of a history of prior preterm birth.

The focus of this review is the current (2020) data on interventions used to reduce the risk of recurrent preterm birth in women with a prior spontaneous preterm birth, as well as risk assessment methods and interventions to reduce the risk of preterm birth in the obstetric population with no history of prior preterm birth.

## DEFINITION OF PRETERM BIRTH

Preterm birth has typically been classified as either spontaneous, which includes preterm labor and premature rupture of membranes, or indicated because of a maternal or fetal complication. Approximately two-thirds of preterm births are spontaneous.^4^ While classification of preterm birth has historically been limited to births that occur between 20 weeks 0 days and 36 weeks 6 days of gestation, many of the etiologies of deliveries at 16 weeks 0 days to 20 weeks are similar to those of later preterm births, and these women appear to have a high risk of recurrent preterm birth.^11,12^ While the true definition of preterm birth applies only to births occurring at 20 weeks of gestation or later, based on the similar etiologies and the high risk for recurrence, preterm birth screening and prevention approaches have been recommended for women with a prior preterm birth as early as 16 weeks’ gestation.^13^

## ETIOLOGIES AND RISK FACTORS FOR PRETERM BIRTH

The strongest predictor of spontaneous preterm birth is a history of spontaneous preterm birth.^8^ Spontaneous preterm birth recurs in 35% to 50% of women, and the probability of spontaneous preterm birth increases with the number of prior spontaneous preterm births.^14^ Several other risk factors have been associated with spontaneous preterm birth, including non-Hispanic Black race, low socioeconomic status, midtrimester cervical length <25 mm, history of dilation and evacuation procedure, maternal smoking, late prenatal care, uterine overdistension, decidual hemorrhage, and short interpregnancy interval.^15-19^

Despite the magnitude of the problem, the underlying etiologies of spontaneous preterm birth remain poorly understood. Strong evidence suggests a genetic contribution to spontaneous preterm birth. Women who were born prematurely are at increased risk to have a spontaneous preterm birth.^20,21^ A further hypothesis suggests that differences in risk-predisposing genetic variant frequencies may partly explain differences in rates of preterm birth among women of different races and ethnicities.^22-25^

## MANAGEMENT OF SINGLETON GESTATIONS WITHOUT A HISTORY OF PRIOR SPONTANEOUS PRETERM BIRTH

### Cervical Length Assessment

As already noted, the majority of preterm births occur in women who have no history of prior preterm birth—that is, they occur in women who are nulliparous or have had prior term births. Development of short cervix has been associated with subsequent preterm birth, and midtrimester cervical length assessment by transvaginal ultrasound is the best clinical predictor of subsequent spontaneous preterm birth.^26^ The prevalence of short cervix in women without a prior spontaneous preterm birth ranges from 1.7% to 7.9%.^27,28^ Risk stratification with cervical length measurement has, therefore, been suggested to help identify potential candidates for intervention.

A cervical length measurement below the 10th centile for gestational age has been used as an arbitrary cutoff to diagnose short cervix. At 18 to 24 weeks of gestation, the 10th centile corresponds to a cervical length <26 mm.^29^ Women with singleton gestations with a cervical length ≤25 mm at 24 weeks of gestation have a 6-fold increased risk of preterm birth, and the risk is along a continuum with the risk of preterm birth increasing as cervical length decreases.^29^ In one study of unselected pregnant women at 22 to 24 weeks of gestation, only 1.7% had a cervical length <15 mm, but these women accounted for 86% of preterm births at <28 weeks of gestation and 58% of preterm births at <32 weeks of gestation.^28^

The positive and negative predictive values of transvaginal cervical length screening for preterm birth are highly dependent on the population being screened, especially at later gestational ages. The positive predictive value of a cervical length <25 mm in identifying subsequent preterm birth at <34 to <35 weeks is good (as high as 81%) in high-risk cohorts of women with a prior spontaneous preterm birth, but it is only fair in the general obstetric population, ranging from 26.3% to 39.1%.^30-32^ A 2017 observational study of nulliparous women reported that a cervical length cutoff of 25 mm identified only 23.3% of women with spontaneous preterm birth at <37 weeks of gestation.^27^ The specificity of short cervical length is also related to the cutoff used. In an unselected cohort and using a cervical length ≤20 mm, the specificity was 99.9% (95% confidence interval [CI] 99.8-100.0) for preterm birth at <34 weeks, but specificity decreased to 90.1% (95% CI 89.0-91.2) for a cervical length ≤30 mm.^32^ Regardless of the cutoff used, the positive predictive value of short cervix remains relatively low.

Despite these limitations, the finding of short cervical length, irrespective of prior pregnancy history, has been consistently associated with an elevated risk of spontaneous preterm birth. No universal agreement on a threshold for diagnosis of short cervix has been reached; most clinicians use 15, 20, or 25 mm as the threshold at which to offer treatment for women with no history of prior preterm birth. As of 2020, neither the American College of Obstetricians and Gynecologists (ACOG) nor the Society for Maternal-Fetal Medicine (SMFM) recommends a universal cervical length screening for all patients but instead state that cervical length screening can be considered as part of a strategy for preterm birth prevention.^33^

### Treatment for Short Cervix in Singleton Gestation – No History of Preterm Birth

Given the association of cervical shortening with preterm birth, several interventions aimed at reducing the risk of preterm birth have been investigated, including 17-hydroxyprogesterone caproate (17-P), cerclage, pessary, and vaginal progesterone.^34-40^ Studies of pessary and cerclage have produced conflicting results, while 17-P treatment has failed to demonstrate benefit when prescribed for the indication of cervical shortening. Results have, however, been consistently more salutary for vaginal progesterone.

#### 17-Hydroxyprogesterone Caproate

In a large multicenter trial, women with a cervical length <30 mm at 16 to 22 weeks were randomized to receive weekly injections of 17-P or placebo. The rate of preterm birth was similar between groups (25.1% vs 24.2%, relative risk [RR] 1.03, 95% CI 0.79-1.35), and no improvement was seen in neonatal outcomes.^36^ Two smaller studies produced conflicting results on the efficacy of 17-P in the setting of short cervix, with one study demonstrating benefit similar to vaginal progesterone and the other demonstrating no reduction in preterm birth.^41,42^ In aggregate, in the absence of a history of prior preterm birth, 17-P has not been consistently demonstrated to reduce the occurrence of spontaneous preterm birth, even in the setting of short cervical length.

#### Cerclage

Cervical cerclage, an operative procedure in which a suture is placed in purse-string fashion around the cervix, is most traditionally used for the prevention of recurrent cervical insufficiency (painless dilation). Cerclage has historically been reserved for patients with a diagnosis of cervical insufficiency. However, cervical insufficiency is now recognized to be along a spectrum, with some women demonstrating cervical shortening prior to painless dilation or clinically apparent signs such as labor or rupture of membranes.^43^

While compelling data demonstrate the benefit of cerclage in the setting of short cervix among women with a prior preterm birth, in the absence of a history of prior preterm birth, cerclage has not generally been demonstrated to result in a reduction of preterm birth.^44-48^ In a study published in 2004, 253 women with very short midtrimester cervical lengths (<15 mm) were randomized to cerclage (n=127) vs expectant management (n=126); the incidence of preterm birth at <33 weeks’ gestation was not improved with cerclage placement (22% vs 26%, RR 0.84, 95% CI 0.54-1.31).^48^ A meta-analysis of 5 randomized trials that included 419 asymptomatic women with cervical length <25 mm and no prior preterm birth (n=224 in the cerclage group) found that cerclage placement did not prevent preterm birth.^49^ These studies excluded women with visible membranes, and these findings therefore may not extend to this subgroup of women. However, a 2018 retrospective study examined the utility of cerclage placement in women being treated with vaginal progesterone because of short cervix who developed further cervical shortening to <10 mm. The cohort who received vaginal progesterone plus cerclage delivered 7 weeks later than those treated with progesterone alone (34 weeks 3 days vs 27 weeks 2 days, *P*<0.001), and the rate of spontaneous preterm birth was reduced even after controlling for confounders (RR 0.11, 95% CI 0.03-0.41).^50^ Neonatal outcomes, including neonatal intensive care unit admission, respiratory distress syndrome, and necrotizing enterocolitis, were all significantly improved as well. Given the conflicting results in these studies, the role of ultrasound-indicated cerclage placement in women without a history of preterm birth who develop further cervical shortening despite treatment with vaginal progesterone needs additional investigation. Neither the ACOG nor the SMFM provides recommendations for this subgroup of patients.

#### Cervical Pessary

As cervical length screening has become more common, interest has grown in nonpharmacologic and nonsurgical interventions to reduce preterm birth. The Arabin pessary, a silicone ring-shaped pessary that encircles the cervix, has been studied. Putative mechanisms of action include alteration in the uterine-cervical angle, displacement of the weight of the gravid uterus, prevention of opening of the internal cervical os, and protection of the cervical mucus plug.^51,52^

The results of randomized trials investigating whether placement of an Arabin pessary in women with short cervix reduces the risk of preterm birth have been conflicting.^35,53,54^ Goya and colleagues randomized 385 women with singleton gestations and a cervical length ≤25 mm to Arabin pessary or usual care between 18 and 22 weeks of gestation. Women treated with a pessary had an 82% reduction in preterm birth at <34 weeks compared to the usual care group (6% vs 27%, RR 0.18, 95% CI 0.08-0.37), as well as a similar reduction in adverse neonatal outcomes (3% vs 16%, RR 0.14, 95% CI 0.04-0.39).^35^ In a study funded by the Fetal Medicine Foundation, 932 women with a cervical length ≤25 mm were randomized to pessary or usual care.^53^ Cervical length was measured at 20 weeks 0 days to 24 weeks 6 days. Among the women enrolled in the trial, 16.5% had a history of prior preterm birth. Women in both treatment groups who had a cervical length ≤15 mm were prescribed 200 mg vaginal progesterone nightly (pessary, 43.9%; usual care, 46.9%). No reduction in spontaneous preterm birth was seen at <34 weeks (12.0 vs 10.8%, *P*=0.57, odds ratio [OR] 1.12, 95% CI 0.75-1.69), and no difference was noted in adverse neonatal outcomes (6.7% vs 5.7%, *P*=0.55) with pessary placement compared to usual care.

A 2017 meta-analysis of study-level data on more than 1,400 women, many of whom were included in the previously discussed trials, concluded that for singleton pregnancies in women with a cervical length <25 mm, placement of an Arabin pessary does not reduce the rate of preterm birth at <34 weeks or improve neonatal outcomes.^55^ However, subsequent to the publication of that meta-analysis, the report of a single-center trial from Italy was published. In that trial, 300 women with no history of preterm birth and a cervical length ≤25 mm at 18 weeks 0 days to 23 weeks 6 days were randomized to pessary or usual care.^54^ Women with a cervical length ≤20 mm, nearly 90% of the women in the trial, were also treated with 200 mg vaginal progesterone. Treatment with pessary was associated with a 52% reduction in spontaneous preterm birth at <34 weeks (7.3% vs 15.3%, RR 0.48, 95% CI 0.24-0.95), as well as a reduction in adverse neonatal outcomes (14.7% vs 32.0%, RR 0.46, 95% CI 0.29-0.72). Additional studies investigating the cervical pessary are currently ongoing in the United States and elsewhere, but in 2020, use of a pessary for prevention of preterm birth in women with short cervix remains investigational.

#### Vaginal Progesterone

The efficacy of vaginal progesterone in women with a sonographic diagnosis of short cervix has been demonstrated by 2 multicenter, randomized controlled trials and by independent patient-level meta-analyses that included data from these studies and several smaller trials. Fonseca and colleagues conducted a double-blind trial that randomized women with a cervical length ≤15 mm to 200 mg vaginal progesterone or placebo.^34^ A total of 413 women were treated from 24 to 34 weeks’ gestation. The incidence of delivery prior to 34 weeks was reduced to 19.2% in the group that received vaginal progesterone vs 34.4% in the placebo group (RR 0.56, 95% CI 0.36-0.86). Eighty-five percent of the women included in the Fonseca et al study had no prior history of preterm birth. In a subgroup analysis of these women without a history of preterm birth, a significant reduction in preterm birth rate at <34 weeks was noted in women with short cervix (≤15 mm) who received progesterone (RR 0.57, 95% CI 0.35-0.93).

In the PREGNANT trial, Hassan and colleagues reported that administration of vaginal progesterone gel (90 mg) to women with a cervical length of 10 to 20 mm identified at 19 weeks 0 days to 23 weeks 6 days of gestation resulted in a significant reduction in the rate of preterm birth at <33 weeks of gestation (8.9% vs 16.1%, RR 0.55, 95% CI 0.33-0.92) and at <35 and <32 weeks of gestation.^37^ Moreover, the study demonstrated a neonatal benefit, with a significant reduction in respiratory distress syndrome (RR 0.39, 95% CI 0.17-0.92). Only 16% of the study population had a history of prior preterm birth, and even after excluding these subjects, progesterone was still associated with a significant benefit in the setting of isolated short cervix (RR 0.50, 95% CI 0.27-0.90).

A 2018 meta-analysis incorporated data on 974 singleton gestations with a cervical length ≤25 mm.^38^ Two of the 5 studies included in the meta-analysis specifically enrolled women with short cervix, while the women from the other studies represent the short cervix subgroup enrolled in the total study patient population.^34,37,56-58^ The meta-analysis reported a reduction in the risk of preterm birth at <32 weeks of gestation (RR 0.64, 95% CI 0.48-0.86) with vaginal progesterone treatment; preterm births at <28, <34, and <37 weeks of gestation were significantly reduced as well. In addition, the meta-analysis showed a reduction in composite neonatal morbidity and mortality (RR 0.59, 95% CI 0.38-0.91), as well as a reduction in birthweight <2,500 g and <1,500 g.^38^ This meta-analysis included data on 128 women with a cervical length of 21 to 25 mm; in this subgroup, the reduction in preterm birth was not statistically significant (RR 0.55, 95% CI 0.22-1.38).

While these data are compelling, treatment with vaginal progesterone following the diagnosis of short cervix and the threshold of cervical length at which to initiate treatment remain areas of debate. The US Food and Drug Administration (FDA) did not approve vaginal progesterone for the indication of preterm birth prevention in the setting of short cervix, in part because data from the PREGNANT trial failed to demonstrate a benefit when only US patients were analyzed. In addition, the FDA declined approval because vaginal progesterone did not appear to be effective in Black or obese women. Despite debate about the clinical utility in all subgroups, given the data on the potential benefit and lack of harm, the ACOG and SMFM have recommended vaginal progesterone as a management option for women with short cervix.^33^ In addition, with evidence of the benefits of vaginal progesterone administration in the setting of short cervix and its cost-effectiveness, some authors have recommended universal cervical length screening for asymptomatic women without a prior preterm birth.^59-61^ The cost-effectiveness of such recommendations, however, is founded on a single, not serial, cervical length measurement at the time of the anatomy ultrasound examination. While treatment with vaginal progesterone is not associated with any significant risks, its use for the indication of short cervix is off-label in 2020.

Many formulations of micronized progesterone contain peanut oil in the excipients (the inert materials in which the drug is suspended). Women with severe peanut allergies, such as anaphylaxis, should not receive micronized progesterone capsules.^62^ Vaginal gel formulations do not contain peanut oil and can be used by women with peanut allergies.^63^ Other contraindications to vaginal progesterone treatment include the typical contraindications to hormonal therapy, such as hormone receptor–positive breast cancer.

## MANAGEMENT OF SINGLETON GESTATIONS WITH A HISTORY OF PRIOR SPONTANEOUS PRETERM BIRTH

### Progesterone Treatment

The National Institute of Child Health and Human Development (NICHD) Maternal-Fetal Medicine Units Network conducted a multicenter double-blind randomized controlled trial of 463 women with a singleton pregnancy and prior spontaneous preterm birth between 16 and 36 weeks who received 17-P or placebo. Treatment with 17-P was associated with a 34% reduction in recurrent preterm birth at <37 weeks of gestation (from 54.9% to 36.3%, adjusted RR 0.66, 95% CI 0.54-0.81), as well as significant reductions in early preterm birth at <32 and <35 weeks and significant reductions in infant complications (intraventricular hemorrhage, necrotizing enterocolitis, and need for supplemental oxygen).^64^ In February 2011, the FDA approved 17-P for the prevention of recurrent preterm birth, and use of this agent for prevention of recurrent preterm birth became the standard of care in the United States.

As one of the conditions for market approval, the FDA required the manufacturer of 17-P to conduct a replication trial. The multisite international PROLONG (Progestin's Role in Optimizing Neonatal Gestation) trial used the same study design, inclusion criteria, and exclusion criteria as the previously noted NICHD trial.^65^ A total of 1,708 women with a prior spontaneous preterm birth were enrolled and randomized to either 17-P or placebo. The study showed that 17-P administration did not reduce the rate of preterm birth at <37 weeks of gestation (17-P 11% vs placebo 11.5%, RR 0.95, 95% CI 0.71-1.26), nor did it reduce neonatal morbidity (5.6% vs 5.0%, RR 1.12, 95% CI 0.68-1.61).^66^ This study could not recruit well in the United States because 17-P was already on the market and incorporated into the standard care for women with prior preterm birth, resulting in significant demographic and risk differences between the PROLONG trial and the NICHD study. Only 22% of patients enrolled in PROLONG were from the United States; 61% were from Russia and Ukraine. In addition, only 1.1% of patients had a cervical length <25 mm and only 7% of the patients were Black—two of the greatest risk factors for preterm birth. These differences in study populations and the conflicting results of the 2 trials have introduced considerable uncertainty and controversy into the management of patients with prior preterm birth. In October 2019, the FDA convened an advisory panel to review the data on 17-P. The panel voted 9-7 to recommend that the drug be removed from the market.^67^ In October 2020, the FDA Center for Drug Evaluation and Research proposed withdrawal of 17-P from the market. The final decision is subject to potential additional public hearings and ruling from the FDA Commissioner.^68^

The SMFM has advocated that critical differences between the study populations in the original NICHD trial and the PROLONG study account to some extent for the disparate results. A 2020 statement from SMFM concludes that providers can reasonably continue to use 17-P in women with a risk profile similar to that of the enrollees in the NICHD study.^69^ Given the preponderance of data in a US population, the SMFM states that women with a singleton gestation and a history of prior spontaneous preterm birth between 20 and 36 weeks 6 days of gestation may be prescribed intramuscular administration of 250 mg 17-P weekly, starting at 16 to 20 weeks of gestation until 36 weeks of gestation or delivery. If 17-P is not available or the patient declines the medication, vaginal progesterone may be a reasonable alternative.^69^

The utility of vaginal progesterone has been investigated in women with prior spontaneous preterm birth. In 2003, da Fonseca and colleagues reported the results of a double-blind randomized controlled trial of 142 women at high risk for preterm birth (94% had a prior preterm birth) who were treated with either 100 mg vaginal progesterone per day or placebo.^70^ The incidence of preterm birth was reduced at <37 weeks (28.5% to 13.8%, *P*=0.03) and at <34 weeks of gestation (18.6% to 2.7%, *P*=0.002). While subsequent studies have produced mixed results, a 2019 meta-analysis readdressed the question of the optimal intervention for women with a prior preterm birth and concluded that vaginal progesterone treatment was associated with a reduction in recurrent preterm birth at <34 weeks (OR 0.29, 95% CI 0.12–0.68) and <37 weeks (OR 0.43, 95% CI 0.23–0.74). 17-P also reduced preterm birth at <37 weeks (OR 0.53, 95% CI 0.27–0.95) and neonatal death (OR 0.39, 95% CI 0.16–0.95).^71^ Many of the studies in this meta-analysis were conducted outside of the United States, so these findings may not be generalizable to the US population.

### Cervical Length Screening and Cerclage

Cervical length is inversely related to risk of recurrent preterm birth and gestational age at delivery, and the transvaginal approach offers better sensitivity to detect cervical shortening than the transabdominal approach.^29,72,73^ Traditional history-based cerclages are placed at 12 to 14 weeks’ gestation. Ultrasound-indicated cerclage should be considered for women with a prior spontaneous preterm birth if the cervical length shortens to <25 mm in the midtrimester (16 weeks to 23 weeks 6 days).^44,74^ Although some controversy exists regarding optimal cervical length screening regimens, based on the regimen used in the largest randomized trial that demonstrated benefit, women with a prior spontaneous preterm birth at <34 weeks should begin transvaginal cervical length assessment at 16 weeks, with serial measurement every 2 weeks until 23 weeks.^74^ If cervical length shortens to ≤30 mm during surveillance, the screening interval should be reduced to weekly.^44^

The strongest evidence for benefit of cerclage in the setting of short cervix comes from a meta-analysis^75^ of patient-level data derived from 5 randomized trials enrolling a total of 504 women (n=250 cerclage procedures).^44-48^ Among women with singleton gestations, a prior preterm birth, and sonographic diagnosis of short cervix, cerclage reduced preterm birth at <37 weeks by 30% and reduced composite perinatal morbidity and mortality by 36%. The number of cerclage procedures needed to prevent 1 perinatal death in this group was estimated to be 20.^75^

Furthermore, for women with a history of preterm birth, especially if the diagnosis of cervical insufficiency is questionable, data indicate that they can be safely monitored with transvaginal ultrasound.^76^ For women who do not develop cervical shortening, additional procedures such as cerclage placement may be avoided. Sonographic monitoring with subsequent ultrasound-indicated cerclage in women with a prior preterm birth has equivalent obstetric outcomes to history-indicated cerclage, but only a fraction (36% to 42%) of the monitoring cohort ultimately requires cerclage.^75,76^

### Combination Therapies

Data regarding the efficacy of combining therapies, such as intramuscular progesterone with or without vaginal progesterone and with or without cervical cerclage, are limited. The utility of combined treatment with history-based cerclage and intramuscular 17-P is unclear, and small retrospective studies have reported mixed results.^77,78^ In a secondary analysis of the Owen et al cerclage trial^44^ that evaluated the subgroup of women with short cervix who were also using 17-P, investigators found a trend toward benefit in the cerclage group.^79^ This study was not, however, designed or powered to examine the combination therapy or the therapies’ interactions. Thus, although no additional benefit to cerclage has been reported when cervical shortening occurs in women on 17-P,^79,80^ the SMFM recommends continuation of 17-P in women who receive an ultrasound-indicated cerclage.^81^

## CONCLUSION

Preterm birth remains a major public health problem. Strategies including cervical length screening, 17-P administration, vaginal progesterone administration, and cervical cerclage placement may help reduce the rates of preterm birth in specific patient populations. Development of and adherence to protocols for patient evaluation and risk stratification will help identify patients at highest risk for preterm birth and the optimal targets for therapeutic interventions.

## Notes

This article meets the Accreditation Council for Graduate Medical Education and the American Board of Medical Specialties Maintenance of Certification competencies for Patient Care and Medical Knowledge.
